# Effectiveness of Transitional Care from Hospital to Home in Frail Older Adults: A Systematic Review

**DOI:** 10.1093/geroni/igab046.2995

**Published:** 2021-12-17

**Authors:** Ji Yeon Lee, Yong Sook Yang, Eunhee Cho

**Affiliations:** 1 Yonsei University, Seoul, Seoul-t'ukpyolsi, Republic of Korea; 2 Yonsei University, Kwangju, Kwangju-jikhalsi, Republic of Korea; 3 Yonsei University College of Nursing, Seoul, Seoul-t'ukpyolsi, Republic of Korea

## Abstract

Frail older adults are at high risk of negative consequences from hospitalization and are discharged without completely returning to their pre-existing health status. Transitional care is needed to maintain care continuity from hospital to home. This systematic review aimed to examine transitional care for frail older adults and its effectiveness. The Cochrane guidelines were followed, and search terms were determined by PICO: (P) frail older adults, not disease-specified; (I) transitional care initiated before discharge; (C) usual care; and (O) all health outcomes. Four databases were searched for English-written randomized controlled trials (inception to 2020), and eight trials were ultimately included. Frail older adults in eight trials (1996–2019) totaled 2,785, with a mean sample size of 310. The intervention components varied from hospital care (e.g., geriatric assessment, discharge planning, rehabilitation) to follow-up care after discharge (e.g., home visit, phone follow-up, community service). Most measured outcomes were readmission (n = 7), function (n = 4), quality of life (n = 4), self-rated health (n = 3), and mortality (n = 3). Statistical significance was reported in the following number of trials: readmission (n = 2), function (n = 2), quality of life (n = 1), self-rated health (n = 3), and mortality (n = 0). The effectiveness of the intervention on each outcome was inconsistent across the trials. Varied transitional care between hospital and home was implemented to improve health status; however, its effectiveness was controversial. A novel, yet evidence-based approach is needed to develop transitional care interventions for these vulnerable populations.

